# 3D chromatin architecture and transcription regulation in cancer

**DOI:** 10.1186/s13045-022-01271-x

**Published:** 2022-05-04

**Authors:** Siwei Deng, Yuliang Feng, Siim Pauklin

**Affiliations:** Nuffield Department of Orthopaedics, Rheumatology and Musculoskeletal Sciences, Botnar Research Centre, University of Oxford, Old Road, Headington, Oxford, OX3 7LD UK

**Keywords:** Chromatin architecture, Chromatin 3D topology, Epigenetics, Transcription regulation, Tumorigenesis

## Abstract

Chromatin has distinct three-dimensional (3D) architectures important in key biological processes, such as cell cycle, replication, differentiation, and transcription regulation. In turn, aberrant 3D structures play a vital role in developing abnormalities and diseases such as cancer. This review discusses key 3D chromatin structures (topologically associating domain, lamina-associated domain, and enhancer–promoter interactions) and corresponding structural protein elements mediating 3D chromatin interactions [CCCTC-binding factor, polycomb group protein, cohesin, and Brother of the Regulator of Imprinted Sites (BORIS) protein] with a highlight of their associations with cancer. We also summarise the recent development of technologies and bioinformatics approaches to study the 3D chromatin interactions in gene expression regulation, including crosslinking and proximity ligation methods in the bulk cell population (ChIA-PET and HiChIP) or single-molecule resolution (ChIA-drop), and methods other than proximity ligation, such as GAM, SPRITE, and super-resolution microscopy techniques.

## Background

**Fig. 1:**
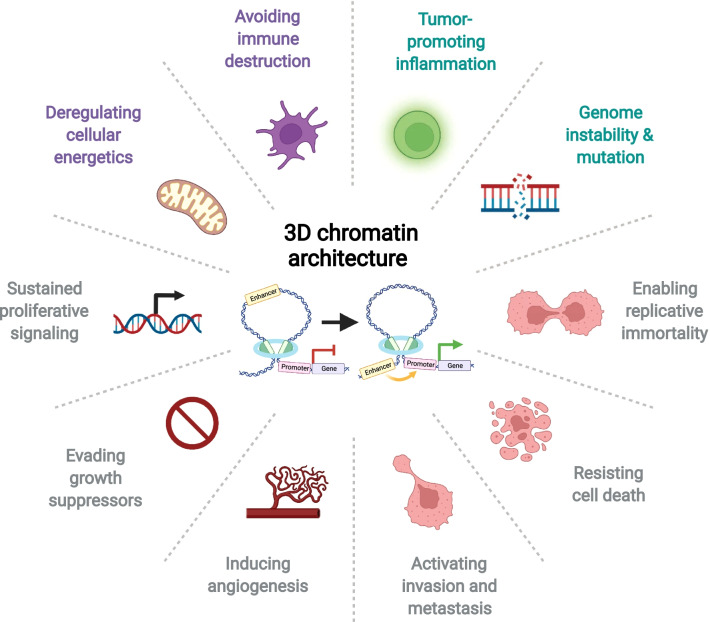
3D chromatin architecture and hallmarks of cancer. The 3D chromatin architecture mediates the induction and repression of genes through multiple levels, including the enhancer–promoter looping that is mediated by transcription factors and structural proteins of chromatin. Therefore, 3D chromatin architecture impacts the hallmarks of cancers: sustaining proliferative signalling, evading growth suppressors, resisting cell death, activating invasion and metastasis, enabling replicative immortality, inducing angiogenesis, reprogramming of energy metabolism, creating the tumour microenvironment, inflammation, evading immune destruction, and genome instability due to mutations

**Fig. 2:**
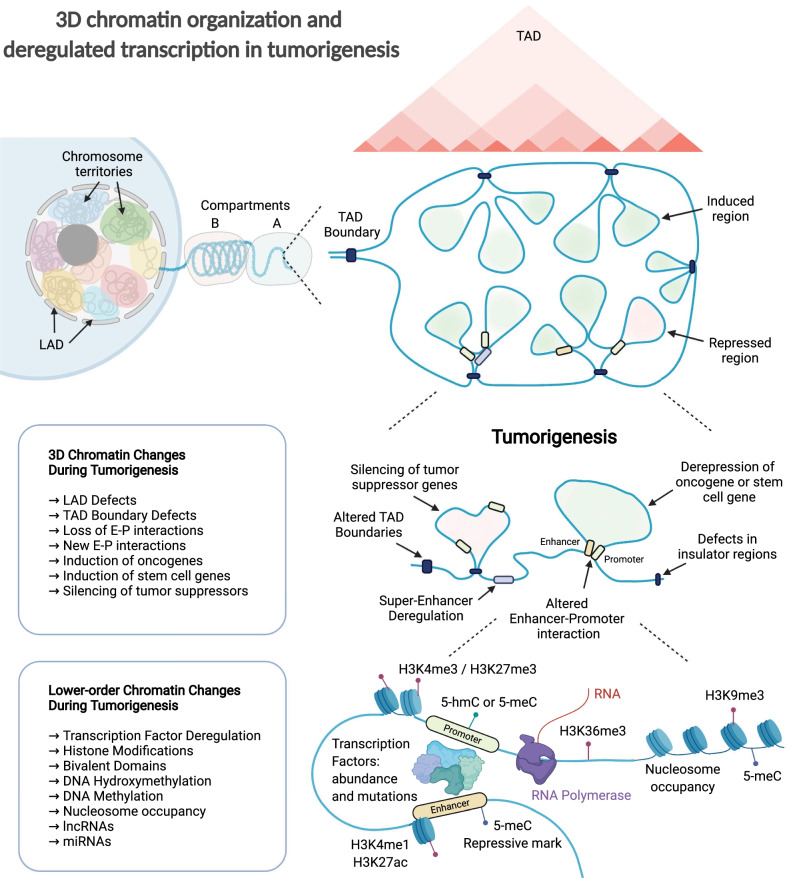
3D chromatin organisation and deregulated transcription in tumorigenesis. Schematic depiction of the different levels of chromatin organisation including chromosome territories in the nucleus, lamina-associated domains (LADs) near the nuclear envelope, A and B compartments corresponding to open and closed chromatin, and a topologically associated domain (TAD) with the 3D chromatin looping in the TADs that can be visualised as chromatin interaction maps (red triangles in TAD). Tumorigenesis involves a range of changes impacting 3D chromatin architecture such as LAD defects, TAD boundary defects, and changes in enhancer–promoter (E–P) interactions regulating gene induction or silencing, as well as lower-order chromatin changes involving transcription factor availability, histone modifications, DNA methylation/hydroxymethylation, nucleosome occupancy, and involvement of long non-coding RNAs (lncRNAs) and micro-RNAs (miRNAs)

**Fig. 3:**
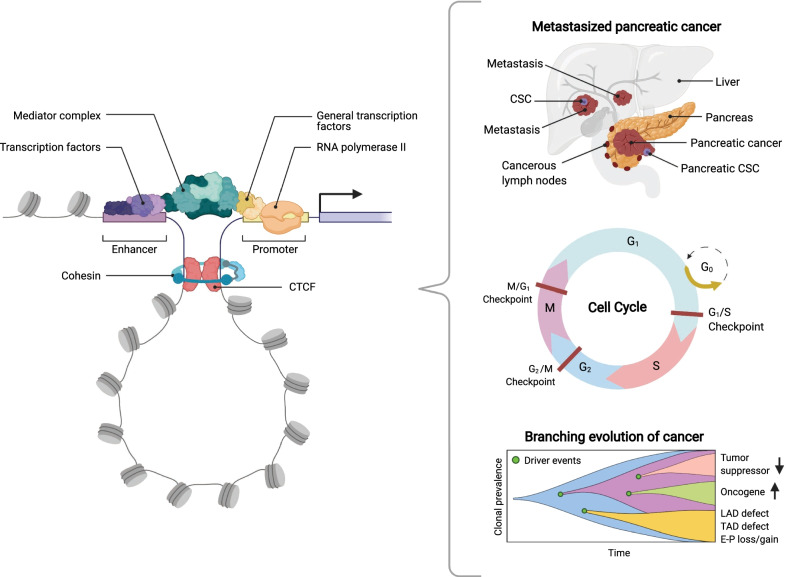
The relevance of 3D chromatin interactions in biological processes. Chromatin looping regulates gene expression in diverse cellular processes. Protein complexes for the chromatin loop bring enhancers in physical contact with promoters and thereby regulate gene expression. The dynamical changes in 3D chromatin architecture are likely to be important for regulating many biological processes during tumorigenesis, including cancer stem cell formation and metastatic processes, the dynamical changes of chromatin during the cell cycle, and the clonal evolution during tumorigenesis that results in different cancer cell characteristics

One of the topological challenges for mammalian cells is to accommodate the large genetic material—about two meters of DNA—in the tiny space of the nucleus: only a few microns in diameter. Meanwhile, cells need to ensure proper biological functions for different cell types in this genetic and epigenetic information [1]. DNA is compacted around histone octamers, namely nucleosome, which is considered the first order of chromatin structure [2, 3]. The higher order of three-dimensional (3D) chromatin structures is various, including *cis*-regulatory interactions (such as enhancer–promoter interaction, or E–P interaction) and repressive interactions (such as Polycomb-mediated interactions and lamina-associated domains, or LADs), mediated by structural elements such as CCCTC-binding factor (CTCF protein) and cohesin [4–8]. These structures have biological implications on the cell cycle, replication, and development and are important in modulating gene function and cell identity [9]. Due to its involvement in the induction and repression of genes through multiple levels, the 3D chromatin architecture has an impact on the hallmarks of cancers (Fig. 1): sustaining proliferative signalling, evading growth suppressors, resisting cell death, activating invasion and metastasis, enabling replicative immortality, inducing angiogenesis, reprogramming of energy metabolism, creating the tumour microenvironment, inflammation, evading immune destruction, and genome instability due to mutations [10]. Curaxins, a class of anti-cancer drugs, have recently been reported to target 3D chromatin architecture in cancer treatment [11, 12], underlining opportunities for exploring therapeutical agents targeting 3D chromatin architectures. This review summarises new aspects of different 3D chromatin architectures and their implications in cancer (Figs. 2, 3). We also highlight the advances in studying 3D chromatin interactions and recently developed bioinformatic tools.

## Types of 3D chromatin architecture

### Topologically associating domain (TAD)

Topologically associating domains (TADs) are self-interacting regions characterised by increased intra-domain interactions [13, 14]. The key player in defining the boundary of TADs is CTCF, a highly conserved zinc-finger DNA-binding protein, detailed in the section below (Fig. 2). TADs are established during mammalian embryogenesis, especially during the four-cell to the eight-cell stage [15, 16]. There is a plethora of evidence suggesting the developmental roles of TADs [17], providing their modulatory roles in cell cycle and DNA replication [18–22], even beyond mammals [23].

The dysregulation of TADs is related to various diseases [24], especially developmental malformations and tumorigenesis. Yang et al. [25] found that 75% of hyperdiploid (gain of chromosomes) paediatric acute lymphoblastic leukaemia (ALL) have a loss of TAD insulation. Shortly after, the same research group examined 1418 cases of B-cell precursor ALL (BCP ALL) patients among five patient cohorts to elucidate the pathological impact of somatic hemizygous 13q12.2 microdeletions [26]. They found that 13q12.2 deletion at 5’ of FMS-like tyrosine kinase 3 (*FLT3*) gene disturbs TAD boundaries and enhancers of the *FLT3*, which contributes to leukaemogenesis. TAD also involves various solid tumours. In gastric adenocarcinoma, Ooi et al. [27] demonstrated the mechanism mediated by cyclin E1 (*CCNE1*) reorganisation, by disruption of *CCNE1* gene-associated TAD boundaries and TAD interactions, resulting in *CCNE1* overexpression in primary tumours. In breast cancer, Abdalla et al. [28] showed Eleanor non-coding RNAs (ncRNAs) outlines the TAD borders of the *ESR1* gene locus in the active nuclear compartment of the long-term oestrogen-deprived MCF7 cells, which controls breast cancer cell apoptosis. Wu et al. [29] also showed multiple myeloma is associated with TAD boundaries and size. More recently, Akdemir et al. [30] found that somatic mutation load is in line with TAD boundaries in cancer cells. Taken together, TAD is associated with tumorigenesis.

As described by Huang et al. [8], the potency of CTCF-mediated transcriptional insulation likely depends on several characteristics, including the number of tandem CTCF protein binding sites, DNA sequences surrounding the protein binding motifs, and the location of the CTCF protein binding sites (inside TAD boundaries is more likely to function as an insulator than those outside TAD boundaries). However, further insight is needed into the factors that control the characteristics of the boundary elements. More recently, studies suggested that TAD and TAD boundaries are not limited to cancer but could also contribute to complex trait heritability, especially for immunologic, hematologic, metabolic traits, and rare-disease pathogenesis [31]. In the future, therefore, it is possible to explore potential molecules that regulate TAD boundaries by targeting the CTCF protein, which may have broad applications in both cancers and inherited diseases.

Studies employing high-resolution techniques have revealed fine-scale subdomains within TADs, or chromatin nanodomains (CNDs) [32–36]. At the single-cell level, Bintu et al. [37] found that CNDs persist in cells even though CTCF or cohesin depletion defines the TAD boundaries or assists chromatin intermingling (see below). In another study, Szabo et al. [36] investigated CNDs using Hi-C and high-resolution microscopy technologies, supporting the topological structure of CNDs and suggesting their potential functions. Therefore, it would be interesting to study the potential role of CNDs, especially their possible relationships with E–P interactions, and their implications in the development and human diseases such as cancer [36]. Such methods will help identify the specific characteristics of self-renewing cancer stem cells (CSCs) in primary tumours and the particular 3D chromatin changes that dynamically change upon the differentiation and dedifferentiation of CSCs, or during metastatic processes.

### Lamina-associated domain (LAD)

The nuclear lamina (NL), a fibrous layer mainly made up of V-type intermediate filament proteins called lamins, lines the inner nuclear membrane. LADs are the genomic regions in contact with the NL. Human promoters are activated when moved from their native LAD region to a neutral context in the same cell [38], suggesting that LADs are usually linked to repressive regulation [39]. LADs are abundant in di- and tri-methylated histone H3 lysine 9 (H3K9me2 and H3K9me3), and indeed, genes in LADs are mostly lowly expressed [40, 41]. The genome is divided into A (active) and B (repressive) compartments, and therefore, LADs correspond to transcriptionally repressive B compartments [13, 42, 43]. In addition to the repression mediated by histone methylations [44], histone deacetylation is also related to the repression in LAD-associated genes [45–47]. It is worth noting that some promoters and enhancers, which are intrinsically less sensitive to LAD repression, are still active even though they are inside LADs [38], indicating that promoters and enhancers vary greatly in their sensitivity to LAD chromatin.

LADs have cell cycle and development implementations as the change of spatial organisation mediated by both TADs and LADs coincides with cell fate decisions [48]. In the course of mouse embryonic development, LADs are established before the formation of TADs and depend on the remodelling of histone H3 lysine K4 (H3K4) methylation [49]. During the cell cycle, LADs are quickly re-established after mitosis [20, 50–52]. The relationship between NL and chromatin organisation/dynamics has been reviewed by Ranade et al. [53]. Lamins provide a chromatin association platform to bind histones, active and/or inactive chromatins [54, 55]. During the development of early mouse embryos, LADs are established in the zygote before the activation of the zygotic genome, the TAD establishment, and the consolidation of compartments [15, 16, 49]. Besides the repressive regulation of the LAD-associated genes, the NL itself could also regulate developmental genes in a range of tissues, and its disruption often leads to diseases. For instance, a critical role of LAD architecture in mouse and human brain development as well as brain diseases has been reviewed by Ahanger et al. [56].

Diseases caused by *LMNA* mutation or proteins that interact with lamins are called laminopathies [57], and these are mostly autosomal dominant [58]. Laminopathies caused by mutations in A-type NL may cause rearrangement of genome chromatin structure. (Their mechanisms and pathophysiologies have been reviewed by Briand and Collas [59].) There are two prevailing hypotheses for laminopathies: gene expression hypothesis and structural hypothesis (see review by Liu and Ikegami [60]). The gene expression hypothesis believes mutations interrupt the interactions between NL and LADs, while the structural hypothesis states mutations disrupt the nuclear envelope.

In recent years, the relatedness of laminopathies with cell ageing, cell cycle, and cell fate determination has been widely reported [61–63]. Cellular senescence (cell ageing) is also sometimes related to oncogene activation—this phenomenon is named oncogene-induced senescence (OIS) [64]. OIS cells lose most of their constitutive LADs, suggesting potential relationships between LADs and cancer [64]. The possible mechanisms in this process have been explored in recent decades. The abnormal morphological structure of the cancer cell nucleus may indicate that cancer cells have an atypical NL structure [65, 66]. More recently, Ji et al. [67] found that depletion of TOPORS (TOP1 Binding Arginine/Serine-Rich Protein), a tumour suppressor, reduces chromatin–lamina interactions and the coverage of LADs. Irianto et al. [68] and Kundu et al. [69] have summarised the role of NL in cancer, including cancer cell migration, tumour growth, transcriptional regulations, and epigenetic alternations. Nevertheless, the detailed mechanisms between LADs and cancer still need to be further explored, which might become a potential therapeutical target in future cancer treatment. While the DamID method is used to identify and study LADs [41, 70, 71], technical advancements using it at the single-cell level [72] or methods for investigating NL more directly via live imaging [73] could be utilised for cancer cell heterogeneity. LADs may be variant in the cancer cells due to their cellular heterogeneity [74], and therefore in the future, it is worth investigating whether LAD re-organisation in cancer cells would lead to abnormal gene expression that is crucial to cancer development, cellular dedifferentiation, and the different subpopulations in tumours such as CSCs.

### Enhancer–promoter (E–P) interactions

Enhancers are usually close to the corresponding gene promoters [4, 5]. In the *Drosophila* genome, about 20% of enhancers are located distantly (50–100 kb) from promoters that they activate [75, 76], while the distance in mammals can be larger [4]. Benabdallah et al. [77] reported that decreased E–P spatial proximity is associated with enhancer activation. Enhancers can be classified into three sub-categories based on epigenetic histone modifications: (1) active (methylation on histone H3 lysine 4, or H3K4me1 and acetylation on histone H3 lysine 27, or H3K27ac), (2) neutral/intermediate/primed (H3K4me1), and (3) poised (H3K4me1 and trimethylation on histone H3 lysine 27, or H3K27me3) [78–80]. Active enhancers promote target gene expression, neutral/intermediate/primed enhancers maintain basal levels of gene activation, while poised enhancers are associated with Polycomb-repressive complex 2 (PRC2; detailed below) repression [81]. Poised enhancers can be switched to the active state by removing H3K27me3 and acquiring H3K27ac marks [79, 81]. Enhancers can communicate with the target gene promoters, thereby forming E–P interactions to regulate gene expression [5, 82]. Vice versa, gene expression was also found to regulate E–P interactions: Bas van Steensel and Furlong [83] found that transcripts of genes may conversely regulate the formation of 3D genome architecture and stabilise E–P interactions.

An enhancer that displays increased usage can use multiple promoters [84], suggesting that E–P interactions may have various specificity and selectivity. Studies found that the binding of architecture proteins with enhancers contributes to E–P interaction specificity [85]. Indeed, structural proteins, such as CTCF protein (detailed below), play an important role in constraining E–P interactions, contributing to 3D genome architectures and regulating gene expression [86, 87]. CTCF also contributes to the heterogeneity in gene expression, even in a homogeneous cell population upon responding to stimuli [88]. Enhancer activities can also be positively or negatively regulated by DNA loops formed by Lac repressor [89]. Whalen, Truty, and Pollard [90] suggested that E–P interactions are regulated by various marks—not only structural proteins and epigenetic modifications but also transcription factors (such as Yin Yang 1 (YY1) reported by Weintraub et al. [91]) and transcription.

Enhancers can be transcribed, and the resulting enhancer RNAs (eRNAs) are shown to establish long-range interactions between enhancers and promoters [92, 93]. In addition, enhancer long non-coding RNAs (elncRNAs) are enriched around chromatin loop anchors, reinforcing the corresponding E–P interactions [94]. The eRNAs and their communication between promoters have recently been reviewed by Ray-Jones and Spivakov [95].

Stable E–P interactions have been observed in *Drosophila* embryogenesis [76], indicating that E–P interactions are important in the developmental stages, such as intestinal differentiation [96], cell fate decisions [97, 98], limb formation [99], and the development of mammalian external genitals [100]. Therefore, aberrations in E–P interactions, such as mutations in genes encoding proteins mediating E–P interactions and/or enhancer-binding proteins, are involved in developmental abnormalities and disease such as Cornelia de Lange syndrome (CdLS) [101, 102] (Figs. 2, 3).

E–P interaction is also an important mechanism in cancer development (see review by Feng et al. [103, 104]). ALL is mainly caused by mixed-lineage leukaemia (*MLL*) gene rearrangements, and H3 lysine 79 di- or tri-methylation (H3K79me2/3) is required for transcription factor binding at enhancers in MLL-AF4 leukaemia cells [105]. More recently, Godfrey et al. [106] reported that, in MLL-AF4 leukaemia cells, this epigenetic marker H3K79me2/3 controls E–P interactions and further activates pan-cancer stem cell marker protein PROM1/CD133. ncRNAs also play a role in T-lineage ALL (T-ALL). Yang et al. [107] found that the formation of neo-loops in the non-coding region hijacks enhancers, which regulates the expressions of transcription factors (including TLX3, TAL2, and HOXA) in T-ALL patients. In addition, eRNA *ARIEL* boosts E–P interactions, thereby activating *ARID5B* expression and promoting the TAL1-induced transcription of the *MYC* oncogene [108]. eRNA can also regulate 3D chromatin architecture and, therefore, E–P interactions in solid tumour development (see review by Isoda et al. [109]). In solid tumours, intrigued by more than 100 single nucleotide polymorphism (SNP) risk loci related to colorectal cancer revealed by genome-wide association studies (GWAS), Tian et al. [110, 111] found that these risk loci drive long-range E–P interactions, regulating several oncogenes including ATF1, E2F1, FADS2, and AP002754.2. Chen et al. [112] expanded this approach by evaluating GWAS-identified variants in six major types of cancer, including colorectal, lung, ovary, prostate, pancreas, and melanoma. Among 270 candidate target genes, they found that 180 genes (66.7%) had evidence of *cis*-regulation by risk loci via E–P interactions, further supporting the crucial functional roles of E–P interactions in cancer.

New methods to study the functional roles of E–P interactions have been developed to study their role in development and disease. Wu et al. [113] described an in situ kethoxal-assisted single-stranded DNA sequencing (KAS-seq) approach to rapidly capture genome-wide transcription dynamics and enhancer activity. To visualise E–P interactions more intuitively, live imaging approaches have been developed. Hongtao Chen et al. [114] and Yokoshi et al. [115] described a multi-colour live imaging method to directly visualise long-range E–P interactions and their consequences in transcriptional regulations at the single-cell level in live *Drosophila* embryos. These new methods addressed a fundamental issue from sequencing or imaging from fixed samples, which cannot dynamically characterise the transient interaction from the formation of stable 3D chromatin architectures.

However, the nature of E–P interactions is complex and occurs in a context-specific manner. Many genes can interact with multiple enhancers, especially developmental regulator genes [116–118], and their expression levels are positively correlated with the number of E–P interactions [116, 117, 119]. Besides, genes can interact with more than one regulatory element [120], and multiple activated genes can be topologically clustered [121, 122], which may be co-regulated by a single enhancer [123]. Therefore, the enhancer may not always directly physically contact the promoter, and additional mechanisms with other protein factors mediating the E–P interactions need to be further explored. Also, different E–P interactions may not be equivalent, and the kinetics of E–P interactions could also be investigated in more detail in the future.

## Key structural elements mediating 3D chromatin interactions

### CCCTC-binding factor (CTCF protein)

CTCF protein is a highly conserved key player in helping chromatin fold into 3D structures, and most mammalian TAD borders bind with CTCF [13, 33]. Xiang and Corces [124] reviewed the latest roles of CTCF in regulating 3D chromatin architecture. In addition, CTCF acts as a versatile factor, involving in transcriptional regulation, insulation, and genomic imprinting (see reviews by Merkenschlager and Nora [125], Liu et al. [126], Braccioli and de Wit [127], and Wang et al. [128]). For example, the binding of CTCF to promoters induces long-range enhancer-dependent gene expression [129]. There are some known factors that impact CTCF binding, including epigenetic control (methylation/demethylation), mutations, and polyADP-ribosylation (see review by Liu et al. [126]). Recently, emerging evidence has revealed new functional roles of this factor. CTCF was found to be involved in DNA double-strand break repair and genomic stability (reviewed by Tanwar et al. [130]). Also, Alharbi et al. [131] reported that CTCF could, directly and indirectly, modulate alternative splicing, indicating that CTCF could also regulate transcriptomic complexity. In addition, Xu et al. [132] reported transcriptional control by CTCF via rewiring of genome-wide chromatin accessibility. Taken together, CTCF is an indispensable structural protein, which involves many key biological processes.

CTCF-mediated looping is highly conserved, established during gastrulation [133], suggesting a critical role of CTCF in early embryo development. About one-third of CTCF binding sites have cell type and/or tissue specificity [134], indicating that the genomic location of CTCF may partially change during cell differentiation. Indeed, CTCF residence time regulates 3D chromatin architecture, gene expression, and DNA methylation in pluripotent stem cells [135], suggesting that CTCF could dynamically control gene expression during development. Also, CTCF occupancy can be altered in response to environmental stimuli, such as temperature stress [136]. In addition, Zhang et al. [137] reported CTCF and transcription as a dynamic regulator of 3D chromatin architecture during the mitosis to G1-phase cell cycle. Ren and Zhao [138] have summarised the functional role of CTCF in regulating cellular diversity. Therefore, CTCF binding plays a critical role in regulating differentiation, cell fate decisions, and development (see reviews by Zheng and Xie [139], Arzate-Mejía et al. [87], and Agrawal and Rao [140]).

In recent years, the most extensively studied disease related to CTCF protein is cancer. Debaugny and Skok [141] reviewed the oncogenic roles of CTCF and CTCFL (CTCF-like or BORIS; the paralog of CTCF, see below section for BORIS). Human *IDH* gene mutant gliomas exhibit DNA hypermethylation, which reduces CTCF binding at the *PDGFRA* oncogene TAD border, eliminating TAD border insulation, and activating the *PDGFRA* oncogene [142]. Recent developments have shed further light to CTCF and its role in tumorigenesis. In prostate cancer progression, Alpsoy et al. [143] found that bromodomain-containing 9 (BRD9) interplays with androgen receptor (AR) and CTCF, regulating AR-dependent gene expression. In breast cancer, Wong et al. [144] examined *Nm23-H1* gene expression (a factor that correlates with metastasis). They found that CTCF and early growth response 1 (EGR1) could regulate *Nm23-H1* gene expression, thereby controlling breast cancer cell metastasis. In melanoma, Sivapragasam et al. [145] found that CTCF binding is associated with UV damage formation, facilitating the establishment of mutation hot spots in melanoma cells. Altogether, CTCF mediates a broad range of locus-specific interactions and gene expression regulations in normal cells, and their dysregulation can support pathologies including cancer.

Aberrations in CTCF can lead to human developmental diseases (see review by Lazniewski et al. [146]). A key mechanism is the interruption of *cis*-regulatory interactions, such as E–P interactions, through perturbations of 3D chromatin architecture, which has recently been emphasised [see review by Qiu and Huang [147] in CTCF protein and E–P interactions in leukaemogenesis]. For example, Zhou et al. [148] reported that zinc finger 143 (ZNF143) mediates the interactions between CTCF and E–P loops, which is essential in haematopoietic stem cell and progenitor cell function. Another intriguing area related to CTCF is cell ageing. Recently, Takayama et al. [149] reported that CTCF acts as a gatekeeper that controls quiescent-to-activated transition in human hematopoietic stem cells. Recent developments suggest CTCF could also intervene in the process of senescence by reduced binding, thereby downregulating *POLD1* gene expression [150]. In addition, Miyata et al. [151] found that the pericentromeric ncRNA modulates CTCF binding and inflammatory gene expression in senescence. Therefore, understanding the relationship between CTCF and cell ageing could be a potential approach to restoring the normal function of senescent cells and is also relevant for tumorigenesis.

### Polycomb group (PcG) protein

Polycomb group (PcG) proteins mainly act as transcriptional repressors [6] and are the main mechanisms utilised in facultative heterochromatin [152, 153], which dynamically define cell identities via epigenetic regulation of developmental genes (see review by Entrevan et al. [154]). PcG protein complexes bind to chromatin called Polycomb group response elements, which then recognises the epigenetic mark H3K27me3 and compacts chromatin leading to gene silence [155]. Most PcG proteins are subunits of one of four categories of protein complexes: Polycomb-repressive complexes 1 (PRC1), Polycomb-repressive complexes 2 (PRC2), Pho-repressive complex (PhoRC), and Polycomb-repressive deubiquitinase (PR-DUB) [156], in which PRC1 and PRC2 are most extensively studied. PRC1 has six different complexes (PRC1.1–PRC1.6), and PRC2 exists in two different forms (PRC2.1 and PRC2.2) (see review by Tamburri et al. [157]). PRC1 is central to the Polycomb system because Polycomb-repressive machinery requires PRC1 catalytic activity [158]. PRC1 works by catalysing the mono-ubiquitination of histone H2A at lysine 119 (H2AK119ub1) [159–161]. Also, PRC1 can detect H2AK119ub1 deposition, which is a central hub that gathers PcG repressive machinery to preserve cell transcriptional profiles [157]. More recently, increasing evidence has suggested that PRC1 also demonstrates transcription activator function. (The transcription activation and repression characteristics of PRC1 have been reviewed by Geng and Gao [162].) The other player, PRC2, can bind scaffold proteins SUZ12 and EED, writing mono-, di-, and tri-methylation of histone H3 lysine 27 (H3K27me1, H3K27me2, and H3K27me3) on the chromatin [163–165]. The detailed structure and function of PRC2 have been reviewed by Moritz and Trievel [166]. PRC2 recruitment and H3K27 methylation are crucial in the spatiotemporal regulation of developmental gene expression and cell fate control (see reviews by Laugesen et al. [167] and van Mierlo et al. [168]). Schuettengruber et al. [169] summarised the core components in the PcG complex and their corresponding epigenetic functions.

The key function of PcG proteins is to regulate the 3D chromatin architecture of the target genes involved in cell differentiation and identity (see review by Entrevan, Schuettengruber, and Cavalli [154]). Also, long-range 3D chromatin interactions mediated by Polycomb genome architecture have been observed. Kraft et al. [170] suggested PRC2 can mediate long-range Polycomb-associated DNA contacts spanning tens to hundreds of megabases across multiple TADs. Joshi et al. [171] also observed extremely long-range promoter–promoter interactions mediated by H3K27me3 Polycomb architecture identified by Capture Hi-C.

PRC1 and PRC2 are so important in development because they are required for embryonic stem cell differentiation and embryonic development, and the absence of either PRC1 or PRC2 will lead to developmental failure [172–175]. Emerging evidence suggests that PcG proteins also mediate DNA repair and genome stability (see review by Fitieh et al. [176]). In addition to its developmental and regulatory roles in chromatin architecture and gene expression, studies have shed light on their behaviours in various disease states, including cancer, aiming to explore how it can be targeted pharmacologically [see review by Chan and Morey [177] for the roles of PcG proteins in regulating stem cells and cancers]. One of the key players targeting PRC2 activity in cancer treatment is enhancer of zeste homologue 2 (EZH2), which is an enzymatically active subunit of the PRC2 complex that methylates H3K27 to promote chromatin compaction and transcriptional silencing (see reviews by Margueron and Reinberg [178] and Di Croce and Helin [179]). Disturbance of EZH2 behaviours in cancer includes both gain-of-function and loss-of-function mutations in EZH2, overexpression of EZH2, mutations in the H3K27 demethylase UTX, and mutations in the SWI/SNF chromatin remodelling complex (see review by Kim and Roberts [180])—this is related to cancer initiation, metastasis, immunity, metabolism, and drug resistance (see review by Duan, Du, and Guo [181], Gan et al. [182], and Huang et al. [183]). Tiffen et al. [184] reported EZH2 as a mediator of treatment resistance in melanoma, and Park et al. [185] illustrated EZH2 functions in prostate cancer. A phase I study of EZH2 inhibitor GSK2816126 was completed in 41 patients with advanced hematologic and solid tumours [186]. This study demonstrated that GSK2816126 manifested modest anticancer activity, but it has a relatively short half-life, limiting effective exposure. Another clinical trial on EZH2 inhibitor Tazemetostat has recently been completed in malignant mesothelioma patients, showing similar outcomes [187].

Therefore, the Polycomb-mediated developmental regulation may be worthwhile to be investigated in the future to better understand processes regulating stem cell differentiation or dedifferentiation, the cellular heterogeneity among tumour cells and CSCs. In addition, more clinical trials on novel EZH2 inhibitors are ongoing [181, 188], which should yield more results to deepen our understanding of the clinical potential of this novel treatment avenue. Current EZH2 inhibitors may not be highly effective in certain cancer types. Therefore, in the future, further clinical trials on combination therapies with EZH2 inhibitors will be promising to maximise therapeutic benefits, and the development of highly predictive biomarkers for EZH2 therapeutic response will be needed. Also, novel EZH2 inhibitors targeting EZH2 post-translational modifications may have therapeutic potentials in cancer therapy [189]. Moreover, it has been shown that some natural products can modulate EZH2 activity [190]. Due to toxicity and relatively low efficiency with current EZH2 inhibitors, therefore, developing natural agents to modulate EZH2 activity may be a new approach to reduce side effects in cancer therapy. Beyond EZH2, overexpression of another Polycomb protein in PRC1, chromobox homolog protein 2 (CBX2), is also associated with poor survival by maintaining CSCs, which might be an emerging approach targeting Polycomb proteins that could be used in cancer therapy [191]. Recently, Rosenberg et al. [192] identified RNA motifs relevant to PRC2 binding and its repressive function in mouse embryonic stem cells, which may provide new ideas on PRC2-targeting molecules in disease treatments. In addition, histone H2A mono-ubiquitination (H2Aub) is catalysed by PRC1 and removed by the PcG-repressive deubiquitinase (PR-DUB)/BAP1 complex, and H2Aub deposition can interplay with PRC2-catalysed histone H3K27 methylation—this crosstalk is also involved in cancer pathologies (see review by Barbour et al. [193]). The H2Aub deposition can affect 3D chromatin architecture and therefore could further impact gene expression [193]. However, the regulation of H2Aub deposition is still largely elusive. Therefore, targeting H2Aub deposition or removal, which indirectly modulates PRC2-catalysed histone H3K27 methylation cascade, might be another potential approach in cancer treatment.

### Cohesin

The cohesin protein complex is one of the best-known structural maintenance of chromosomes (SMC) complexes (the structures have been reviewed by Yuen and Gerton [194] and Gligoris and Löwe [195]), which mediates sister chromatid cohesion by holding sister chromatids during cell cycles to ensure appropriate chromosome segregation [196–198], and it is also important in maintaining the 3D architecture of interphase chromosomes [199–201]. After the formation of CTCF anchors, cohesin can then assist the loop extrusion process (Fig. 3), which is mainly classified into three types of mechanisms [7]: diffusion by Brownian motion [202], the extrusion via motor activity from ATP hydrolysis [203], and/or extrusion by pushing cohesin with other translocating factors, possibly via RNA polymerase II (RNAPII) [204]. The extrusion procedure may be a combination of multiple mechanisms [7]. The detailed mechanism of the loop formation and enlargement has been extensively reviewed by Davidson and Peters [205], van Ruiten and Rowland [206], Kamada and Barillà [207], and Sedeño Cacciatore and Rowland [208].

Cohesin protein complex also regulates gene expression through modulating chromatin structures. Recent studies showed that the *N*-terminus of CTCF could directly interact with cohesin in chromatin looping, which can be regulated by several RNA-binding domains (see review by van Ruiten and Rowland [209]). In fact, cohesin and CTCF are interdependent with each other in gene expression regulation: CTCF works in cooperation with other proteins, including cohesin [146]; in turn, CTCF boundaries and enhancers also regulate cohesin loading and loop extrusion [210], and the Myc-associated zinc finger (MAZ) also collaborates with CTCF to regulate cohesin-mediated genome organisation [211]. Cohesin and CTCF separate chromatin into different spatial domains, and the chromatin interactions within domains occur more frequently than between domains. This spatial segmentation therefore results in ‘regulatory segmentation’, where genes can be co-regulated within domains (see review by Merkenschlager and Nora [125]) by facilitating long-range E–P interactions (see reviews by Zhu and Wang [212] and Dorsett [213]).

While being implicated in developmental disorders, cohesin-related protein dysregulation is also associated with tumour initiation and development, and mutations in genes encoding cohesin protein complex have been identified in cancers [see review by De Koninck and Losada [214], and also Waldman [215] summarised the recent insight to cohesin protein complex in cancer pathogenesis. Fisher et al. [216], in particular, summarised the cohesin mutations in myeloid malignancies], where the hinge domain in the cohesin complex has been shown to play a critical role [217]. In vertebrate somatic cells, there are two types of cohesin subunit protein, SA1 and SA2 (encoded by *STAG1* and *STAG2* genes), which hold SMC3, SMC1, and RAD21 proteins to form the cohesin protein complex, called cohesin-STAG1 and cohesin-STAG2 [218]. *STAG2* is the most frequently mutated in solid cancers [219, 220], and in hematologic malignancies, genes including *STAG1*, *STAG2*, *RAD21*, *SMC1A*, and *SMC3* are frequently mutated [221–223]. Also, precocious dissociation of sisters 5 (PDS5) is an associated protein of cohesin complex [224]. It has been shown that PDS5B protein behaves as a tumour suppressor, as *PDS5B* gene expression level is reduced in gastric and colorectal cancers [225], and about 47% of breast cancer have low expression of *PDS5B* [226]. The low *PDS5B* expression may be regulated by epigenetic modifications, especially methylation, of CpG island of *PDS5B* promoter [226], and PDS5B reduction might promote cancer cell proliferation through the IL-6/STAT3/cyclin D axis [227]. In addition, *miRNA-223* microRNA could interact with *PDS5B* mRNA, and inducing *PDS5B* expression or using *miRNA-223* inhibitor could inhibit pancreatic cancer cell growth [228]. Therefore, in the future, targeting dysfunctional or mutated cohesin complex can be a novel strategy in cancer therapies, and cohesin mutated patients can be considered as an individual subgroup in clinical trials. Also, the abnormal expression of cohesin complex genes is associated with cancer development [229], which could be used as novel prognostic tools in cancer.

Therefore, the cohesin complex has versatile functions in many disorders, and could open therapeutic possibilities in the future as a molecular target. Cohesin protein complex can interact with other architectural proteins, including CTCF and PcG protein [230], which may echo its fundamental role in 3D chromatin structure and gene regulations. Antony et al. [231] reviewed the potential therapeutical targets regarding cohesin complex mutations in cancer. More recently, in hematologic malignancies, including MDS and acute myeloid leukaemia, in particular, mutations in the cohesin complex have been reported to be the key driver to alter DNA damage repair and chromatin architecture [232], providing more therapeutic opportunities in blood cancers. Also, DNA breakages at CTCF/cohesin protein-binding sites could be a robust early detection tool of blood cancer-susceptible individuals in the future [233]. Moreover, USP13 deubiquitinase was recently found as a novel cohesin-interacting protein regulating ubiquitination, which might provide novel strategies in regulating cohesin protein levels in human diseases. Finally, in the future, a more detailed understanding of the cohesin complex will be gained by more advanced technologies and methods, such as super-resolution visualisation [234] and liquid chromatin Hi-C [235]. For example, previously, STAG1 and STAG2 subunits of cohesin were considered to behave similarly, while recent studies have discussed the differences between these two types to better understand their regulatory roles (see review by Cuadrado and Losada [236]). The deeper understanding will enable exploring more therapeutical approaches in disease treatment.

### Chromatin remodeller

Chromatin remodellers are multi-protein complexes. They have ATPase activity, using energy from ATP hydrolysis, to translocate nucleosomes [237], thereby altering chromatin structure and controlling chromatin accessibility [238, 239]. A specialised chromatin domain, the ‘epigenetic reader domain’, senses external signals such as histone modifications in the remodellers [238, 240] to enable other non-catalytic subunits guide the remodeller to dedicated nucleosome positions [238]. Based on the conserved catalytic subunit containing ATPase activity, chromatin remodellers can be mainly categorised into four families, including SWI/SNF (SWItch/Sucrose Non-Fermentable), CHD (Chromodomain-Helicase-DNA binding), ISWI (Imitation SWItch), and INO80 (inositol requiring 80), and they have different epigenetic reader domains (see review by Längst et al. [241]).

Chromatin remodellers have important implications in cancers (see review by Biegel et al. [242]). A cancer genome sequencing project containing 4623 various cancer samples showed the SWI/SNF family has tumorigenesis (tumour suppressor) functions [243], in which about 20% of tumour samples have at least one mutation in the SWI/SNF complex. (The well-known p53 tumour suppressor gene is mutated in about 26% of tumour samples.) Some cancers have reported unexpectedly higher SWI/SNF mutation rates, such as ovarian clear cell carcinoma (75%), clear cell renal cell carcinoma (57%), hepatocellular carcinoma (40%), gastric cancer (36%), and melanoma (34%) [244]. Some mutations in the subunits of SWI/SNF complex have proved to be associated with cancers, although the detailed mechanisms are not yet fully understood. For example, SMARCA4 (BRG1) and SMARCA2 (BRM) are commonly mutated subunits in cancers [244, 245]. Also, another subunit SMARCAD1 has proved to be related to breast cancer migration, invasion, and metastasis [246]. [Tong et al. [247] reviewed the biological mechanisms (especially DNA damage repair) mediated by SMARCAD1.] *ARID2* (AT-rich Interactive Domain 2) genes also encode subunits of SWI/SNF remodellers, which is associated with hepatocellular carcinoma (see review by Loesch et al. [248]).

Since SWI/SNF complex is important in carcinogenesis, recent research tried to investigate its detailed mechanisms and explore possibilities for druggable targets. Bayona-Feliu et al. [249] reported SWI/SNF maintains genome integrity and stability via R-loop-dependent transcription–replication conflicts. This study indicates mutations of SWI/SNF complex trigger carcinogenesis via inducing genome instability. Based on this hypothesis, a recently developed bromodomain inhibitor was proposed, targeting SWI/SNF complex to promote double-strand break and DNA repair [250], which demonstrates the feasibility for targeting SWI/SNF in cancer chemotherapy. Hong et al. [251] reported carcinogenetic mechanisms of SMARCB1 and liver cancer.

The interactions between different chromatin remodeller families and also other structural proteins are not clear, and how they may cooperate in gene expression regulation. It has been recently reported that SWI/SNF can cooperate with ISWI to regulate transcription in yeast and mice [252], but similar mechanisms may need to be validated in human cells. Chang et al. [253] observed increased occupancy of ACTL6A within SWI/SNF complex in human squamous cell carcinoma, which neutralises polycomb-mediated repressions. As SWI/SNF complex has a typical epigenetic reader domain, it is worth investigating the interplay between epigenetic-modifying enzymes and SWI/SNF complex in cancers. A recent study reported evidence of interactions between SWI/SNF ATPase subunit SMARCA2 and histone methyltransferase NSD2 in multiple myeloma development [254]. In addition, mutations in SWI/SNF complex may mediate response and resistance to cancer immunotherapy and resistance. For example, *ARID2* has been shown to be associated with the immune blockade in melanoma [255]. Also, in pancreatic ductal adenocarcinoma, *ARID1A* mutation and *B2M* inactivation can be related to metastasis and immunotherapy resistance [256]. Therefore, targets associated with SWI/SNF complex may be beneficial to immunotherapy outcomes.

### Extrachromosomal DNA (ecDNA)

Extrachromosomal DNA (ecDNA) is a double-strand DNA molecule without centromeres and telomeres outside of the chromosome, usually 1–3 Mb in length [257]. The formation of ecDNA may be a combination of several processes, including replication slippage, episome formation, DNA double-strand-break based events, rolling, translocation–excision–deletion–amplification, and chromothripsis (see reviews by Gu et al. [258] and Wang et al. [259]). Its behaviours in cancers have drawn great attention in recent years, involving important aspects in tumorigenesis, including tumour heterogeneity, oncogene amplification, drug resistance, and senescence (see reviews by Wang et al. [259] and Qiu et al. [260]).

One of the important mechanisms of ecDNA in controlling oncogene expressions is through ‘hijacking’ enhancers. Morton et al. [261] reported that *EGFR* involved ecDNA amplicon patterns using a computational approach in primary human glioblastoma specimens, and discovered ecDNA adjacent enhancers are consistently co-selected with the corresponding oncogenes during ecDNA biogenesis. They also revealed distal chromatin contact during the formation of ecDNA as the co-selected enhancers could also be located outside of the TAD of the original chromosome demonstrated by the chromosome conformation capture technique. Later, Helmsauer et al. [262] observed that extrachromosomal circular *MYCN* amplicons in neuroblastoma are consistently co-selected with proximal local enhancers, which may not be endogenous local enhancers. Therefore, the ecDNA could acquire abnormal enhancer activity to drive oncogene expressions that promote tumorigenesis.

To further investigate 3D chromatin architecture and long-range chromatin interactions on ecDNA, recently, Zhu et al. [263] leveraged ChIA-PET and ChIA-Drop interaction assays and found that ecDNA can act as a highly mobile super-enhancer element that drives gene expressions in glioblastoma and prostate cancer cell lines. Also, most recently, Hung et al. [264] reported that ecDNA hubs (clusters of 10 – 100 ecDNA in the nucleus) drive intermolecular E–P interactions to induce oncogene expression. Therefore, it would be interesting to interfere ecDNA hubs as a novel future cancer therapeutic strategy.

### Brother of the Regulator of Imprinted Sites (BORIS)

Brother of the Regulator of Imprinted Sites (BORIS) protein is a paralog to CTCF, also known as CTCFL [265]. BORIS protein is mainly associated with spermatogenesis but also cancer formation. Pugacheva et al. [266] characterised 23 isoforms in germline and cancer cells, varying in zinc-fingers DNA-binding domain, amino and carboxyl termini, and expression levels. These isoforms may have different regulating abilities and transcriptional regulation outcomes [267–271].

BORIS is essential for spermatogenesis [272–274]. There are two categories of CTCF/BORIS-bound regions: single CTCF target sites (1xCTSes) that are bound by CTCF alone and double CTCF target sites (2xCTSes) either bound by both CTCF and BORIS or BORIS alone in germ cells and BORIS-positive somatic cancer cells [275]. BORIS is overexpressed in several cancers (see reviews by Klenova et al. [265] and Martin-Kleiner [276]) and is involved in important biological processes in cancer [277, 278], such as the apoptosis pathway [279]. Debruyne et al. [280] reported molecular mechanisms mediated by BORIS that promote chromatin interactions in cancer cells. BORIS recently drew more attention as a target in CSCs [281], suggesting new immunotherapeutic avenues in treating advanced metastatic and drug-resistant cancers. For example, BORIS induces *OCT4* overexpression through histone methylation, promoting CSC-like characteristics in liver cancer cells [282].

### Phase separation

Phase separation forms when intracellular proteins and/or nucleic acids build a functionally specialised non-membrane-bound (or ‘membraneless’) local subcompartments, which can be either liquids, solids, or gels (see review by Boeynaems et al. [283]). The protein components in these subcompartments can be either scaffolds or reactors [284], where scaffolds drive the dynamic phase separation (such as FUS family proteins), and reactors mediate specific biological reactions. Nucleic acid components in phase separation are mainly non-coding RNAs, especially lncRNAs [285].

Among them, liquid–liquid (or gel-like) phase separation has been widely studied in recent years and sheds light on its role in tumorigenesis [284]. For example, lncRNA TUG1 could bind with other structural proteins such as methylated PC2 protein CBX4 inducing liquid–liquid phase separation and present genes to PcG clusters to regulate growth signals [285]. The spatiotemporal interactions between protein and nucleic acid are weak interactions controlled by dynamic post-translational modifications (such as phosphorylation, acetylation, and methylation) and RNA modifications (such as m^6^A modification) [286]. In cancer cells, the functional phase separation can condensate cancer-related proteins, involved in genomic instability, transcriptional regulation of cancer-related proteins/pathways, and protein degradation (see reviews by Jiang et al. and Wang et al., who summarised the oncogenic processes involved in phase separation [286, 287]).

Some recent work has brought new insights on the mechanisms of phase separation in carcinogenesis and its possibility as a novel therapeutical strategy in cancer. Mechanistically, fusion genes in cancers may promote tumorigenesis via phase separation. Ahn et al. [288] recently deciphered how liquid–liquid phase separation forms unstructured intrinsically disordered regions (IDR) contributing to leukaemia. They found that a homeodomain-containing transcription factor chimaera, namely NUP98-HOXA9, is essential for forming liquid–liquid phase separation. Therefore, it promotes the establishment of super-enhancer, thereby activating leukaemogenic genes. Similarly, in lung cancer, Qin et al. [289] reported EML4-ALK fusion aggregates through phase separation in various cancer cell lines. This fusion protein activates downstream STAT3-mediated signalling pathways, thereby promoting tumorigenesis. Another work suggested that phase separation is associated with glycogen accumulation especially in liver cancer [290]. The researchers found that glycogen phase separation in the cytoplasm in early-stage liver tumours causes the accumulation in glycogen, which blocks the Hippo signalling pathway and drives tumorigenesis. Therefore, it is possible to monitor glycogen changes for early diagnosis and/or prognosis of cancer. Nevertheless, a clear understanding of how phase separation drives cancers remains elusive. Recent advances in the methods for studying phase separations (see review by Mehta et al. [291]) help in the deeper investigation of the molecular function and mechanisms involved in this process.

## Advances in studying 3D chromatin interactions

### Chromatin interaction analysis with paired-end tag (ChIA-PET) sequencing

From chromosome conformation capture (3C), chromosome conformation capture-on-chip (4C), chromosome conformation capture carbon copy (5C), to Hi-C, chromatin conformation capture techniques lack specificity to a specific protein [13, 33, 51, 292, 293]. To improve the specificity, Fullwood et al. [294] proposed chromatin interaction analysis with paired-end tags (ChIA-PET), which implements enrichment strategies and can identify both short- and long-distance 3D chromatin interactions mediated by a specific protein of interest at the whole-genome scale. The protocol has been updated from short PETs (2 × 20 bp) to longer PETs up to 2 × 250 bp [295], which increase its mapping efficiency and accuracy. It has been extensively applied to study 3D chromatin interactions in human/mouse cells mediated by CTCF and RNAPII [91, 98, 296–298].

**Fig. 4:**
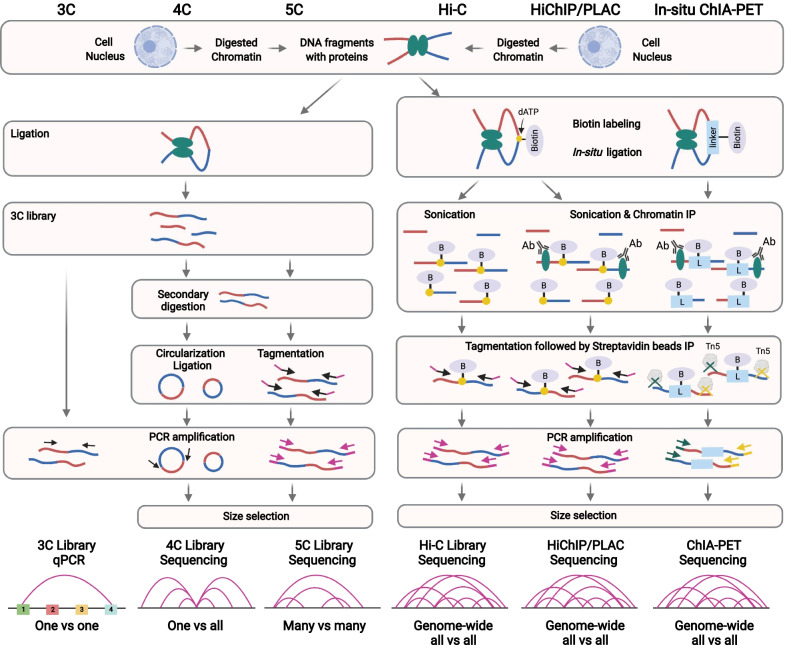
Comparison of 3D chromatin analyses methods. Comparison of the main experimental steps of 3C, 4C, 5C, Hi-C, HiChIP/PLAC, and ChIA-PET that allow identifying chromatin interactions between enhancers and promoters as well as chromatin domains

To yield the required number of informative reads, the number of cells required for each ChIA-PET experiment has been large [298]. To address this problem, in situ ChIA-PET was introduced by Bertolini et al. [299], where the proximity ligation is performed in intact nuclei (Fig. 4). This method has higher efficiency in capturing intra-molecular interactions and requires fewer cells to detect protein-mediated chromatin interactions [299].

### HiChIP

Another method to address the cell number issue of the ChIA-PET experiment is HiChIP [300], which takes advantage of in situ Hi-C [33] and transposase-mediated library construction approach [301]. HiChIP is performed by stabilising chromatin contacts in situ in the nucleus to reduce false-positive interactions and improve chromatin interaction capture efficiency [302]. Then, ChIP is performed to enrich the interactions associated with the protein of interest before high-throughput sequencing [300]. Due to data type, some tools can be used for both ChIA-PET and HiChIP data processing (Table 1).

**Table 1:** Summary of advanced methods studying 3D chromatin interactions and related bioinformatic tools

Technologies	Tools	Comments
ChIA-PET [294, 295]	ChIA-PET Tool: Li et al. [303] ChiaSig: Paulsen et al. [304] MICC: He et al. [305] Mango (also for HiChIP): Phanstiel et al. [306] ChIA-PET2: Li et al. [307] ChIAPoP: Huang et al. [308] ChIA-PET Tool V3: Li et al. [309] ChIA-PIPE (also for HiChIP): Lee et al. [310]	ChIA-PET tool is the first software package designed for ChIA-PET data analysis ChiaSig and MICC were developed later, which uses statistical models to adjust random noise Mango is a bias-correcting pipeline based on statistical confidence, which also corrects bias caused by non-specific interactions due to genomic proximity Since ChIA-PET tool and Mango are only compatible for half-linker data in the linker trimming step, and ChiaSig and MICC are only a step in the analysis pipeline, ChIA-PET2 was developed, which supports both half-linker and bridge linker data, and integrates all steps required for the analysis ChIAPoP, which is another fully automated pipeline integrated all the above features and claimed to outperform the above tools ChIA-PET tool has updated to ChIA-PET tool V3 for updated experimental protocol ChIA-PIPE is the most comprehensive fully automatic pipeline that integrates many features
HiChIP [300]	hichipper: Lareau and Aryee [311] MAPS: Juric et al. [312] HiC-Pro: Servant et al. [313] Fit-HiC: Ay et al. [314] Juicer: Rao et al. [33]; Durand et al. [315] HiChIP-Peaks: Shi et al. [316] FitHiChIP (also for ChIA-PET): Bhattacharyya et al. [317] cLoops (also for ChIA-PET): Cao et al. [318] Peakachu (also for ChIA-PET): Salameh et al. [319] AQuA-HiChIP: Gryder et al. [320] HiC-DC + : Sahin et al. [321]	ChIA-PIPE used for ChIA-PET data analyses can also be used for HiChIP data analysis Hichipper and MAPS are designed specifically for HiChIP data processing One can also use HiC-Pro pipeline for HiChIP data processing, and perform contact calling using Fit-HiC, Mango, and Juicer HiChIP-Peaks is a peak calling algorithm, which generate satisfactory results for HiChIP data and discover loops FitHiChIP is a loop calling method, which can also perform differential HiChIP analysis for characterising differential loops cLoops is another loop calling method using statistical model Peakachu deploys a random forest classification framework to predict loops AQuA-HiChIP can perform differential chromatin interaction analysis between samples
ChIA-drop [322]; GAM [323]; SPRITE [324]	ChIA-DropBox (ChIA-Drop): Tian et al. [325] MATCHA (ChIA-Drop and SPRITE): Zhang and Ma [326] MIA-Sig (ChIA-Drop, GAM, and SPRITE): Kim et al. [327]	

### ChIA-drop

The above methods only reflect pairwise and population-level views of chromatin interactions. Zheng et al. [322] reported a method called ChIA-Drop that can reveal the chromatin interactions in single-molecule resolution. It takes advantage of the droplet-based genomic analysis [328] to isolate single chromatin complexes without ligation. ChIA-DropBox [325] was designed specifically for ChIA-Drop data analysis (Table 1). This method makes it possible to characterise the multiplex chromatin interactions (e.g. transcription hub) at a single-molecule view and examine the cellular heterogeneity of chromatin contacts.

### Move away from proximity ligation to genome-wide 3D chromatin interactions

Beagrie et al. [323] developed the genome architecture mapping (GAM) method, the first genome-wide method for capturing 3D chromatin interactions without proximity ligation. GAM measures 3D chromatin distances using ultrathin cryosectioning and DNA sequencing. It infers chromatin spatial organisation by determining the presence or absence of all genomic loci in many random-orientated individual thin nuclear slices from a population of nuclei, which can be analysed by MIA-Sig [327] (Table 1). More recently, the same research group has optimised the GAM protocol (‘multiplex-GAM’) into a faster and more affordable version [329] that could generate informative enrichment contact information using only a few hundred cells.

Split-pool recognition of interactions by tag extension, or SPRITE, can also detect genome-wide 3D chromatin interactions without proximity ligation, developed by Quinodoz et al. [324]. It can identify extremely long-range inter-chromosomal interactions and measure DNA and RNA interactions simultaneously. This research group recently improved SPRITE in the single-cell resolution (scSPRITE), which is more efficient and affordable than the single-cell HiC method [330]. Tools such as MATCHA [326] and MIA-Sig [327] can be used to process both SPRITE and ChIA-drop data (Table 1).

Super-resolution microscopy can be used to visualise 3D chromatin interactions more directly and intuitively. Recently, Su et al. [331] reported a genome-scale imaging technology to visualise 3D chromatin interactions in situ of a single cell. This method utilised a multi-scale approach combining fluorescence in situ hybridisation (FISH) [332] and clustered regularly interspaced short palindromic repeats (CRISPR) labelling methods, which can image more than one thousand genomic loci concurrently in single cells. However, this method depends on the choice of genomic loci, and it is unclear whether the experimental procedures would perturb the chromatin structure (e.g. FISH, CRISPR labelling, and cell fixation).

### Single-cell technologies in 3D chromatin architecture

To study 3D chromatin architecture at the single-cell level, till now, there are two main techniques: imaging-based techniques (especially fluorescence in situ hybridisation of DNA, or DNA-FISH, at the single-cell level) and high-throughput sequencing-based techniques (especially single-cell Hi-C). DNA-FISH-based imaging is a method that can literally observe the chromatin structure at the single-cell level, such as 3D-FISH [333] and cryo-FISH [334]. However, the DNA-FISH-based imaging approaches have limited throughput, which only allows observing a small number of genomic loci at a time.

The recent development of single-cell and next-generation sequencing technologies has opened a new chapter in studying 3D chromatin architecture at single-cell resolution. Single-cell Hi-C is one of the widely used methods which uses Hi-C library preparation in isolated single nuclei [335]. In 2017, Stevens et al. [336] described the first genome-wide 3D interactions in mouse embryonic stem cells using single-cell Hi-C. GAM, as we describe in the above section, can also examine chromatin interactions at single-cell resolution by performing nucleus cryosectioning and sequencing of DNA on nuclear slices [323]. Future efforts (such as reducing sectioning thickness) might be able to additionally increase its resolution. HiCAR (high-throughput chromosome conformation capture on Accessible regulatory DNA) is a most recently developed method that simultaneously investigates the transcriptome, accessible regulatory elements and their interactions, which represents the functional output of chromatin structure and accessibility [337]. HiCAR requires much less input material (can as little as 30,000 cells) than traditional techniques (such as HiChIP [300], PLAC-seq [338], and in situ ChIA-PET [299]).

## Conclusions and future perspectives

In summary, 3D chromatin interactions, including TAD, LAD, E–P interactions, and Polycomb domain, are crucial in transcription regulations, which plays a key role in development and diseases. It is possible to use crosslinking and proximity ligation methods in the bulk cell population (ChIA-PET, HiChIP) or single-molecule resolution (ChIA-drop) to study 3D chromatin interactions. Methods other than proximity ligation, such as GAM, SPRITE, and super-resolution microscopy techniques, are also reported for studying genome-wide 3D chromatin interactions. Bioinformatic tools have been extensively developed to analyse the data. More studies are needed to investigate reducing the number of cells to generate informative data and reduce experimental perturbations of chromatin structure. Also, it would be interesting to combine different analyses in single cells such as single-cell RNA-seq and single-cell ATAC-seq with 3D chromatin architecture. This would widen the technical opportunities to investigate early stages of cancer formation, cell cycle progression, clonal evolution of cancer, chemoresistance and metastatic processes, ultimately combining temporal and spatial dimensions. Integration of single-cell ATAC-seq and 3D chromatin interaction data has been applied to dissect the causal regulatory variants in neurological diseases [339]. Single-cell RNA-seq would reveal the transcriptomic responses in the heterogeneous cell population, and single-cell ATAC-seq could explain the concordance between the chromatin accessibility and the expression profiles. Therefore, these single-cell techniques could cross-validate the scientific findings and provide a broader picture of studying chromatin regulatory behaviours. Ultimately, these understandings revealed by the state-of-the-art technologies will certainly open a new area of research on anticancer therapies. While inhibitors of epigenetic regulatory enzymes exist, only curaxin CBL0137 has been so far reported to target 3D genome by affecting long-range *cis*-regulatory elements via interacting CTCF-binding sites [11, 12]. It is expected that future research in this area will lead to further improved strategies for the treatment of cancer.

## Data Availability

All materials are available within this publication.

## Abbreviations

3D: Three-dimensional
E–P: T interaction enhancer–promoter interaction
LADs: Lamina-associated domains
CTCF: CCCTC-binding factor
TADs: Topologically associating domains
ALL: Acute lymphoblastic leukaemia
BCP ALL: B-cell precursor acute lymphoblastic leukaemia
FLT3: FMS-like tyrosine kinase 3
CCNE1: Cyclin E1
ncRNAs: Non-coding RNAs
CNDs: Chromatin nanodomains
CSCs: Cancer stem cells
NL: Nuclear lamina
H3K4: Histone H3 lysine K4
OIS: Oncogene-induced senescence
H3K4me1: Methylation on histone H3 lysine 4
H3K27ac: Acetylation on histone H3 lysine 27
H3K27me3: Histone H3 lysine 27
PRC2: Polycomb-repressive complex 2
YY1: Yin Yang 1
eRNAs: Enhancer RNAs
lncRNAs: Long non-coding RNAs
elncRNAs: Enhancer long non-coding RNAs
CdLS: Cornelia de Lange syndrome
MLL: Mixed-lineage leukaemia
T-ALL: T-lineage acute lymphoblastic leukaemia
SNP: Single nucleotide polymorphism
GWAS: Genome-wide association studies
KAS-seq: Kethoxal-assisted single-stranded DNA sequencing
BRD9: Bromodomain-containing 9
AR: Androgen receptor
EGR1: Early growth response 1
ZNF143: Zinc finger 143
PcG: Polycomb group
PRC1: Polycomb-repressive complexes 1
PRC2: Polycomb-repressive complexes 2
PhoRC: Pho-repressive complex
PR-DUB: Polycomb-repressive deubiquitinase
H2AK119ub1: Mono-ubiquitination of histone H2A at lysine 119
EZH2: Zeste homologue 2
H2Aub: Histone H2A mono-ubiquitination
SMC: Structural maintenance of chromosomes
MAZ: Myc-associated zinc finger
PDS5: Precocious dissociation of sisters 5
MDS: Myelodysplastic syndromes
BORIS: Brother of the Regulator of Imprinted Sites
1xCTSes: Single CTCF target sites
2xCTSes: Double CTCF target sites
ChIA-PET: Chromatin interaction analysis with paired-end tags
GAM: Genome architecture mapping
SPRITE: Split-pool recognition of interactions by tag extension
FISH: Fluorescence in situ hybridisation
CRISPR: Clustered regularly interspaced short palindromic repeats
SWI/SNF: Switch/sucrose non-fermentable
CHD: Chromodomain-helicase-DNA binding
ISWI: Imitation switch
INO80: Inositol requiring 80
ecDNA: Extrachromosomal DNA
IDR: Intrinsically disordered regions
HiCAR: High-throughput chromosome conformation capture on accessible regulatory DNA
